# Kidney of Pregnancy

**Published:** 1895-01

**Authors:** 


### Selection.

Kidney of Pregnancy.—Tranteroth (*British Medical Journal*) finds that in about half the cases of pregnancy in healthy women, primiparæ or otherwise, a trifling amount of albuminuria is to be detected in the second half of pregnancy. In a minority of cases this symptom, if not due to renal changes; in the majority it represents a special morbid condition, best termed "the kidney of pregnancy." As a rule this condition involves no symptoms besides changes in the kidney. Eclampsia and edema are rare. The pregnancy kidney never changes into the kidney of any chronic form of nephritis. There is no true nephritis of pregnancy. Albuminuria is the rule during labor, especially in primiparæ, and casts (usually hyaline) are to be found in almost a third of the cases. In the albuminuria of pregnancy casts are much rarer. The albuminuria of labor is most marked during the period of dilatation and disappears rapidly during childbed, except when there is fever; later on towards the second week, albuminuria usually indicates catarrh of the lower part of the urinary tract. The albuminuria of pregnancy and labor does not render chloroform dangerous. Renal disease, existing before pregnancy, is greatly aggravated by that condition, often ending in death of the ovum and abortion, after which the disease abates more or less. The causes of pregnancy kidney are the increase of intra-abdominal pressure, changes in the nutrition of the kidney brought about by the altered condition of the blood, and in special cases obstruction of the left ovarian vein which joins the left renal, and compression of the ureter by the fetal head. The last two causes apply to the kidney of labor (*Geburtsniere*), where also septic changes from pieces of fetal appendages play a part. The degree of the changes which make up the kidney of pregnancy depends on the resisting power of that organ in the individual patient.—*Maryland Medical Journal.—Kansas Medical Journal.*

